# Supplementary material for: Biodiversity of rolB/C-like Natural Transgene in the Genus Vaccinium L. and Its Application for Phylogenetic Studies
Source: Int J Mol Sci. 2023 Apr 8;24(8):6932. doi: 10.3390/ijms24086932 (PMC10138537; doi:10.3390/ijms24086932)
Supplement: Supplementary file 1 [file ijms-24-06932-s001.zip › Supplementary Figures (1).pdf]

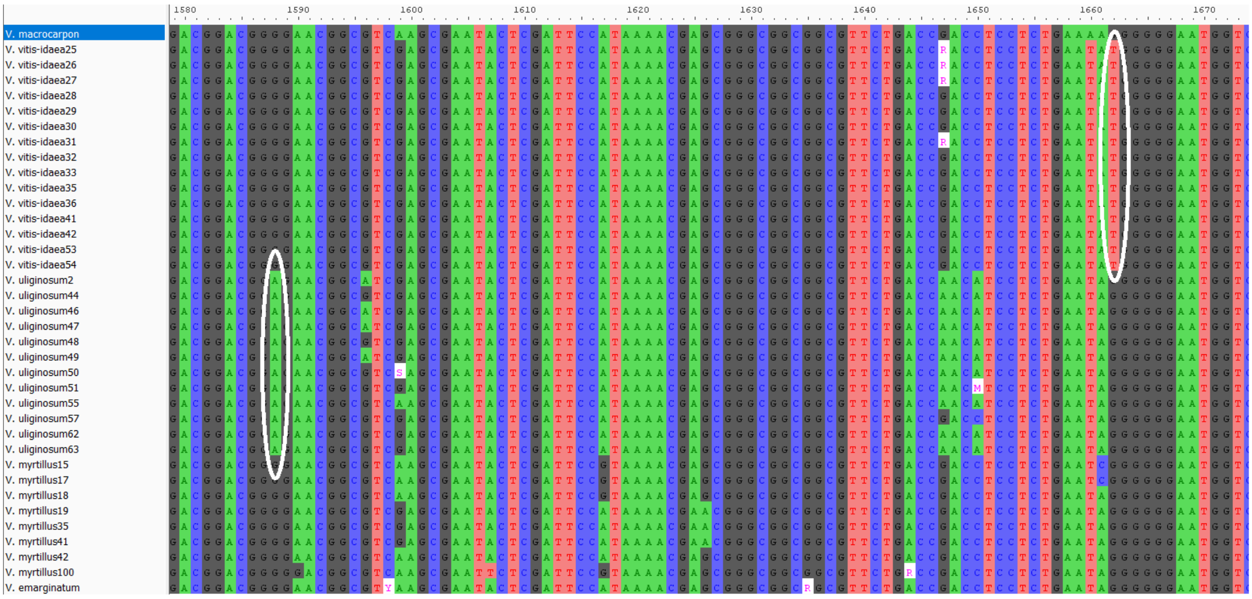

Figure S1. Examples of SNPs specific to *V. uliginosum* and *V. vitis-idaea*. Numbering relative to reference (JOTO01169953.1)

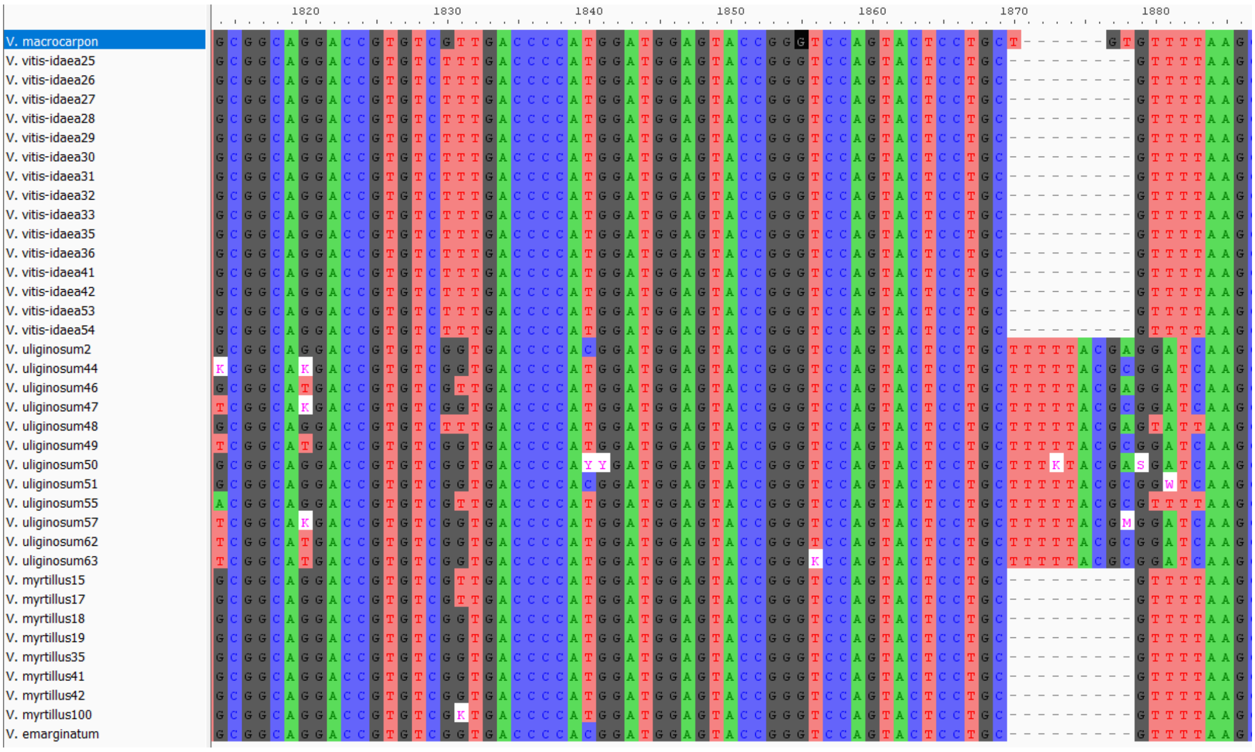

Figure S2. Examples of indels that are multiples of three, typical for different species *Vaccinium*. Numbering relative to reference (JOTO01169953.1)
